# Evidence on Non-Invasive Respiratory Support During Flexible Bronchoscopy: A Narrative Review

**DOI:** 10.3390/jcm14186658

**Published:** 2025-09-22

**Authors:** María Hidalgo Sánchez, Manel Luján, Sergio Alcolea Batres, Julia Álvarez del Vayo, Pablo Mariscal-Aguilar, Carlos Carpio, Rodolfo Álvarez-Sala Walther

**Affiliations:** 1 Pulmonology Department, Hospital Universitario La Paz-Cantoblanco-Carlos III, Faculty of Medicine, Universidad Autónoma de Madrid (UAM), IdiPAZ, 28046 Madrid, Spain; mhidalgosanchez@salud.madrid.org (M.H.S.); sergio.alcolea@salud.madrid.org (S.A.B.); pablo.mariscal@salud.madrid.org (P.M.-A.); rodolfo.alvarezsala@salud.madrid.org (R.Á.-S.W.); 2 Pulmonology Department, Hospital Universitari Parc Taulí, 08208 Sabadell, Spain; mlujan@tauli.cat; 3 Anaesthesiology and Intensive Care Department, Hospital Universitario La Paz-Cantoblanco-Carlos III, 28046 Madrid, Spain; julia.alvarezvayo@salud.madrid.org

**Keywords:** bronchoscopy, CPAP, high-flow therapy, non-invasive respiratory support, oxygen therapy

## Abstract

**Background:** Flexible bronchoscopy (FB) is a widely used diagnostic and therapeutic procedure in patients with pulmonary disease, many of whom are at risk of gas exchange impairment. FB may exacerbate hypoxaemia due to increased airway resistance, alveolar derecruitment, and haemodynamic fluctuations. **Objectives:** To assess the effectiveness of non-invasive respiratory support strategies in preventing oxygen desaturation and respiratory complications during FB. **Methods:** A systematic review and meta-analysis were conducted using PubMed and Cochrane databases, covering studies from 2000 to 2024. Inclusion criteria focused on adult patients undergoing FB with any form of non-invasive oxygen support. Twelve high-quality studies were selected, including randomised trials and prospective cohorts. **Results:** High-flow therapy (HFT) was more effective than conventional oxygen therapy (COT) in maintaining oxygenation and reducing procedure interruptions, especially in patients with moderate hypoxaemia or risk factors such as obesity and obstructive sleep apnoea. Continuous positive airway pressure (CPAP) and non-invasive ventilation (NIV) offered superior oxygenation and ventilatory support in patients with more severe respiratory or cardiac compromise. **Conclusions:** Non-invasive respiratory support should be individualised based on patient risk and procedural complexity. HFT benefits mild-to-moderate cases, while CPAP or NIV is preferable in more severe conditions. Further multicentre randomised trials are needed to establish formal guidelines.

## 1. Introduction

FB is a diagnostic and/or therapeutic procedure frequently performed in patients with pulmonary diseases. Most individuals undergoing this procedure have underlying conditions that impair gas exchange, including pneumonia, interstitial lung disease, or lung cancer, which may result in the development or exacerbation of respiratory failure.

During the procedure, the partial pressure of oxygen in arterial blood (PaO_2_) may decrease by more than 10–20 mmHg, thereby elevating the risk of respiratory insufficiency. Consequently, some form of respiratory support is often required to prevent desaturation episodes. The bronchoscope occupies approximately 10% of the tracheal cross-sectional area and 15% of the cricoid ring. This increases airway resistance, elevating the work of breathing and potentially causing dynamic hyperinflation with an augmented functional residual capacity. When suction is applied, airway and alveolar pressures can fall to zero or even become negative, thereby reducing end-expiratory lung volume, promoting alveolar derecruitment and atelectasis, and ultimately impairing gas exchange [1,2].

These effects are even more pronounced during flexible bronchoscopy with bronchoalveolar lavage (BAL), as aliquots of sterile saline are instilled and only partially retrieved, leaving a substantial volume within the alveoli. This significantly reduces the end-expiratory volume of the affected lung parenchyma below the functional residual capacity [1,2], which may precipitate alveolar collapse and ventilation-perfusion mismatch [3,4]. Although such alterations are typically reversible following bronchoscopy, recovery may require anywhere from several minutes to several hours in patients with severe pulmonary disease.

Bronchoscopy also produces indirect haemodynamic effects. The increase in airway resistance and respiratory effort modifies intrathoracic pressures, which may compromise venous return and afterload, thereby reducing cardiac output. However, sympathetic activation typically results in an approximate 50% rise in cardiac output, which returns to baseline within 15 min following the procedure [5]. Electrocardiographic abnormalities have been reported in up to 21% of patients aged over 60 years [6,7].

Consequently, bronchoscopy represents a substantial cardiopulmonary risk in patients with respiratory or cardiac vulnerability, emphasising the need for an individualised oxygenation or respiratory support strategy.

## 2. Materials and Methods

A comprehensive systematic search was undertaken in the PubMed and Cochrane databases, covering the period from 2000 to 2024. The search employed the following keywords: Bronchoscop and Respiratory Support, Bronchoscop and Oxygen, Bronchoscop and Non Invasive. Only studies published in English and involving adult human participants receiving one or more modalities of oxygen support during flexible bronchoscopy, for either diagnostic or interventional purposes, were considered. No limitations were imposed regarding the type of bronchoscopic procedure or the degree of anaesthetic risk.

**Figure 1:**
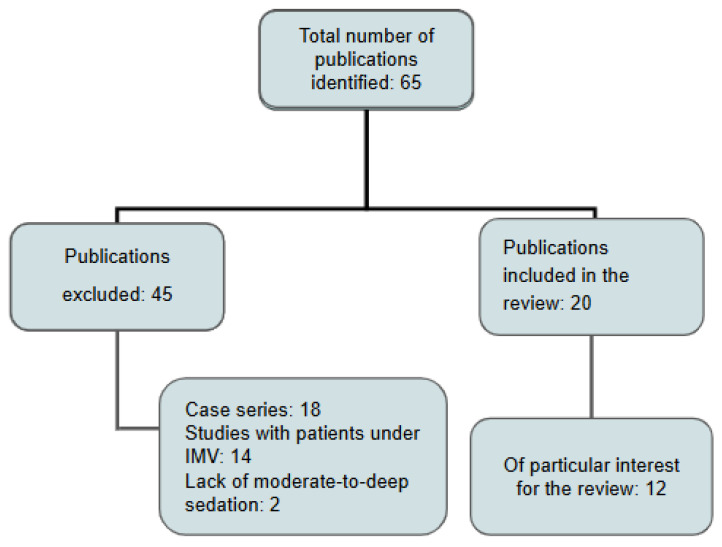
Flowchart for study selection: invasive mechanical ventilation (IMV).

The studies that met the inclusion criteria consisted of randomised controlled trials, prospective and retrospective cohort studies, systematic reviews, and meta-analyses published in peer-reviewed, indexed journals between 2000 and 2024 (Figure 1). From these, twelve studies were selected for in-depth analysis based on their superior methodological quality, larger sample sizes, direct comparison of respiratory support modalities, and publication in high-impact medical journals (Table 1).

Exclusion criteria included articles published in languages other than English, case reports or case series lacking control groups, cross-sectional studies, procedures performed without moderate to deep sedation, animal studies, paediatric populations, and studies involving rigid bronchoscopy, upper airway interventions, tracheal pathology, or invasive mechanical ventilation.

Results are presented in a narrative synthesis, with interpretation and discussion of the published evidence.

## 3. Results

The main findings of the included trials are summarised in Table 1.

### 3.1. Modalities of Respiratory Support During Flexible Bronchoscopy

During flexible bronchoscopy, several respiratory support modalities are employed. COT delivers low-flow oxygen via nasal cannulae, simple masks, or Venturi masks.

HFT provides heated and humidified air/oxygen mixtures at flows up to 60 L/min, with an inspired oxygen fraction ranging from 21 to 100%. CPAP maintains positive end-expiratory pressure (PEEP) throughout the respiratory cycle via a mask, while NIV delivers ventilatory support through inspiratory and expiratory positive airway pressures (IPAP and EPAP). Currently, no formal consensus or clinical guidelines exist regarding the optimal respiratory support strategy during flexible bronchoscopy.

This study provides a narrative review of the current literature from PubMed and Cochrane, with the aim of outlining the respiratory support modalities most frequently used in clinical practice and offering recommendations adapted to patients’ specific clinical profiles.

#### 3.1.1. Conventional Oxygen Therapy

COT consists of delivering a fixed flow of oxygen, which blends with ambient air to satisfy the patient’s inspiratory flow requirements. Its main advantages include accessibility, low cost, and simplicity of use; however, a key limitation is the variability and unpredictability of the fraction of inspired oxygen (FiO_2_) actually delivered. This variability can render COT inadequate in cases of severe hypoxaemia and is highly dependent on the patient’s breathing pattern. Nevertheless, COT remains the most frequently employed form of respiratory support in bronchoscopy settings.

When a patient’s peak inspiratory flow exceeds the oxygen flow provided by conventional systems, FiO_2_ becomes unpredictable [20,21]. Oxygen can be administered via nasal cannulae, simple masks with or without reservoirs, or Venturi masks. Nasal cannulae may be positioned either nasally or orally, with no significant difference in mean peripheral oxygen saturation (SpO_2_) observed during the procedure between these approaches [22]. Several studies have shown that COT offers advantages over no oxygen supplementation in patients undergoing FB who are at heightened risk of desaturation, including those with baseline SpO_2_ < 93%, obstructive ventilatory defects, or forced expiratory volume in one second (FEV_1_) below 1 litre [23]. Additional benefits have been documented in patients with chronic obstructive pulmonary disease and immunosuppression, who face a higher likelihood of requiring intubation within 24 h post-procedure [24]. Furthermore, COT is typically required during BAL or cytological brushing [8,25]. Accordingly, while not universally mandatory, COT is primarily recommended for patients at risk of desaturation—particularly those with baseline SpO_2_ below 93%—and during procedures involving BAL or brushing [26].

Nevertheless, owing to the rising number of severely ill patients with acute or chronic respiratory failure, advanced pulmonary disease, and cardiac comorbidities, conventional oxygen therapy frequently proves insufficient to maintain adequate oxygenation and prevent hypercapnia.

#### 3.1.2. High-Flow Oxygen Therapy

High-flow therapy (HFT) has been adopted in clinical practice as an effective alternative to COT for respiratory support during bronchoscopy. Its principal advantage lies in providing a more stable FiO_2_ at flow rates of up to 60 L/min, which can accommodate or exceed the patient’s elevated inspiratory demand during and after the procedure.

Secondly, HFT produces a modest PEEP within the airways, influenced by flow rate, upper airway anatomy, the patient’s nasal or oral breathing pattern, and the cannula-to-nare size ratio. This generates alveolar distension that improves end-expiratory lung volume and oxygenation in patients with diverse forms of acute respiratory failure (ARF) [27,28]. Thirdly, HFT reduces anatomical dead space in the upper airway, thereby enhancing alveolar ventilation. Finally, it decreases the patient’s work of breathing and lowers upper airway resistance.

HFT promotes a CO_2_ washout from the pharyngeal dead space, which is proportional to the applied flow; each 1 L/min increment in flow corresponds to an approximate 1.8 mL/s increase in clearance within the nasal cavity. This washout effect is also influenced by respiratory rate, becoming more pronounced at lower rates owing to longer expiratory times [29]. Consequently, this mechanism reduces the patient’s respiratory effort compared with COT.

HFT has been effectively utilised to prevent the worsening of ARF during bronchoscopy.

Enhancements in SpO_2_ following the procedure, along with reductions in mucosal trauma and patient discomfort, have been reported, which are attributed to the delivery of warmed and humidified gas.

Moreover, HFT has been demonstrated to decrease episodes of oxygen desaturation and the requirement for airway interventions, such as jaw-thrust manoeuvres or bag-mask ventilation, in patients at risk of hypoxaemia undergoing flexible bronchoscopy under deep sedation, including individuals with obstructive sleep apnoea (OSA), male sex, obesity, advanced age, or hypertension [9,30].

Several studies have compared HFT with other oxygen delivery methods, particularly COT. In a randomised controlled trial involving lung transplant recipients undergoing flexible bronchoscopy for transbronchial biopsy, procedural interruptions occurred more frequently with low-flow nasal oxygen than with HFT [10]. Compared with Venturi masks, HFT at 60 L/min provided superior oxygenation, whereas no significant difference was observed between HFT and Venturi masks at 40 L/min [31]. Another randomised trial examining HFT during endobronchial ultrasound (EBUS) under conscious sedation in patients without acute respiratory failure found no significant reduction in intraprocedural desaturation, and procedure duration did not affect the outcomes [32].

During BAL, the efficacy and safety of HFT compared with COT have been assessed in patients with ARF. No significant adverse events, including endotracheal intubation within 24 h post-procedure, were reported, nor were there notable differences between groups in transient hypoxaemia, fever, or hypotension. HFT enhanced gas exchange during BAL by preserving end-expiratory lung volume and reducing diaphragmatic workload [33,34].

In a cohort of 40 patients undergoing flexible bronchoscopy via the oral route for suspected pneumonia requiring bronchial aspirate collection, HFT at 40 L/min was associated with smaller decreases in SpO_2_ and a lower incidence of desaturation compared with standard oxygen therapy [11].

More recent studies and meta-analyses have questioned the superiority of HFT over COT in improving oxygen saturation, minimising desaturation episodes, and preventing procedural interruptions [12]. The advantages of HFT seem most pronounced in patients with lower baseline oxygenation.

No significant effects on hypercapnia or the incidence of endotracheal intubation (ETI) have been observed. During flexible bronchoscopy with BAL, HFT appears more effective than COT in maintaining oxygen saturation in both ambulatory patients and hypoxaemic individuals in intensive care, without influencing ETI rates [35,36,37].

In certain populations, including lung transplant recipients, or during procedures such as EBUS or foreign body removal, HFT also seems more effective than COT in preventing hypoxaemia. Ben-Menachem et al. [13] compared HFT with COT during flexible bronchoscopy for transbronchial biopsy in lung transplant patients, reporting fewer mild-to-moderate desaturation episodes and reduced procedural interruptions with HFT.

In summary, the advantages of HFT over COT are most evident in patients with significant baseline hypoxaemia, lung transplant recipients, and those at heightened risk of hypoxaemia during flexible bronchoscopy under deep sedation—such as individuals with obstructive sleep apnea (OSA), male sex, obesity, advanced age, or hypertension—particularly when BAL or EBUS is performed. In these patients, HFT improves oxygen saturation, reduces desaturation events, minimizes procedural interruptions, and lessens the need for supplementary airway interventions, without affecting hypercapnia or the incidence of ETI.

#### 3.1.3. Continuous Positive Airway Pressure

CPAP delivers a constant positive pressure throughout the respiratory cycle, promoting the recruitment of atelectatic lung regions, decreasing intrapulmonary shunting, and lowering the patient’s inspiratory effort. Its main limitation is the potential for poor tolerance of the interface and the requirement for specialised masks equipped with accessory ports to allow passage of the bronchoscope.

The main effects of CPAP include enhanced oxygenation through the reduction in intrapulmonary shunting and ventilation–perfusion mismatch, alongside a decrease in the work of breathing. CPAP delivered via a face mask has been shown to effectively recruit alveoli and improve gas exchange in hypoxaemic patients. In those with heart failure, additional benefits have been described, including redistribution of pulmonary oedema, improved left ventricular performance, and reduced respiratory effort.

The application of CPAP via a face mask during bronchoscopy has been assessed in patients with hypoxaemic acute respiratory failure (PaO_2_/FiO_2_ < 300 mmHg) by Maitre et al. [14]. Compared with conventional oxygen therapy, CPAP maintained higher SpO_2_ levels both during and after the procedure, and a smaller proportion of patients required additional respiratory support within six hours post-procedure. During BAL performed as part of flexible bronchoscopy, patients receiving oxygen therapy alone experienced a more pronounced decline in PaO_2_, highlighting the adverse impact of BAL on gas exchange in hypoxaemic individuals. By contrast, no significant reduction in PaO_2_ was observed in the CPAP group, supporting its potential benefit when BAL is indicated. Consequently, CPAP appears particularly advantageous for patients with moderate to severe hypoxaemia undergoing diagnostic procedures that markedly reduce PaO_2_, such as BAL.

#### 3.1.4. Non-Invasive Mechanical Ventilation

NIV provides the benefits of CPAP while additionally delivering inspiratory pressure support, which diminishes the patient’s respiratory effort and maintains adequate ventilation during deeper levels of sedation. Its limitations, beyond discomfort associated with the interface, include challenges in achieving effective patient–ventilator synchrony due to leaks and interference from airway manipulation during the procedure.

Extensive literature and clinical guidelines indicate that NIV preserves normal minute ventilation in patients with impaired respiratory drive, prevents peripheral airway collapse and atelectasis, promotes alveolar recruitment, enhances vital capacity and pulmonary compliance, and decreases the work of breathing. NIV thus allows bronchoscopy to be performed safely for both diagnostic and therapeutic purposes in patients experiencing acute exacerbations of respiratory failure, as well as in those with moderate to severe chronic respiratory insufficiency [38,39,40].

NIV delivered via face mask was first applied during BAL in flexible bronchoscopy in 1996. In a cohort of immunocompromised patients with suspected pneumonia and severe hypoxaemia (PaO_2_/FiO_2_ < 100 mmHg), Antonelli et al. [15] reported that NIV was well tolerated, improved gas exchange, and prevented the need for intubation. Subsequently, its use via face mask was evaluated in immunocompetent patients with hypoxaemic acute respiratory failure of varying severity (PaO_2_/FiO_2_ < 200 mmHg) in comparison with conventional oxygen therapy. NIV maintained optimal gas exchange during and after bronchoscopy without causing haemodynamic instability, was associated with a low incidence of minor complications, and only a small proportion of patients required intubation within eight hours post-procedure. Moreover, NIV has been applied in more complex procedures, including transbronchial lung biopsy and interventional techniques such as balloon dilation, electrocautery, and argon plasma coagulation, with similar efficacy observed in patients with chronic and acute-on-chronic respiratory failure [41,42].

To evaluate the feasibility and safety of NIV during bronchoscopy in patients with chronic obstructive pulmonary disease (COPD) for whom spontaneous ventilation is contraindicated, Da Conceisao et al. [16] conducted a prospective, non-comparative clinical trial. NIV was found to be safe, maintaining adequate gas exchange in hypoxaemic and hypercapnic COPD patients undergoing BAL. SpO_2_ improved significantly during the procedure (from 91 ± 4.7 to 97 ± 1.7%) without falling below 90%. No significant changes in PaCO_2_ or PaO_2_ were observed during the hour following bronchoscopy, no complications occurred, and NIV was well tolerated, effectively reducing the risk of intubation within the first 24 h.

More recently, in 2023, Sharma VK et al. [43] conducted a randomised, controlled, three-arm trial including 90 COPD patients who were randomly assigned (1:1:1) to receive COT, HFT, or NIV. The study evaluated the incidence of hypoxia, nadir SpO_2_, electrocardiographic changes, and vital signs. Participants comprised moderate (n = 51), severe (n = 34), and very severe (n = 5) COPD according to Global Initiative for Chronic Obstructive Lung Disease (GOLD) criteria. SpO_2_ during bronchoscopy was lowest in the COT group (COT: 87.03 ± 5.7% vs. HFT: 95.57 ± 5.0% vs. NIV: 97.40 ± 1.6%, *p* < 0.001). Secondary outcomes were generally comparable, except for respiratory rate, which was higher in the COT group (COT: 20.23 ± 3.1 vs. HFT: 18.57 ± 4.1 vs. NIV: 16.80 ± 1.9, *p* < 0.001). Post-bronchoscopy arterial oxygen tension was greatest in the NIV group (NIV: 84.27 ± 21.6 mmHg vs. HFT: 69.03 ± 13.6 mmHg vs. COT: 69.30 ± 11.9 mmHg, *p* < 0.001), whereas arterial carbon dioxide tension did not differ significantly between groups. The authors concluded that both NIV and HFT are superior to COT in preventing hypoxia during bronchoscopy; however, NIV is associated with reduced patient tolerance and greater procedural complexity for the operator.

Attempts have been made to identify predictive thresholds for complications during bronchoscopy performed with NIV. Reported predictors include PaO_2_ of 69 ± 18.5 mmHg and PaCO_2_ of 49 ± 9.0 mmHg (*p* < 0.05), leukocyte counts of 10.0 ± 4.81 and 14.4 ± 3.10 (*p* < 0.05), and the presence of left heart disease (*p* = 0.046). At present, there are no absolute contraindications to NIV during bronchoscopy; however, it is not recommended for patients who are inherently unsuitable for NIV, such as those with severe acute hypoxaemic respiratory failure and a PaO_2_/FiO_2_ ratio < 150 mmHg. Precise thresholds for hypoxaemia and hypercapnia, as well as risk factors, ventilator settings, indications, and contraindications, require definition and validation in future prospective, multicentre studies [17].

Growing evidence supporting the use of HFT and NIV has prompted comparative studies in patients with mild to moderate hypoxaemic acute respiratory failure. Compared with HFT, NIV improved oxygenation before, during, and after bronchoscopy, and reduced the incidence of desaturation episodes below 90%, without affecting mortality or the need for intubation and invasive mechanical ventilation. Overall, NIV and HFT exhibited comparable efficacy in preventing hypoxaemia during bronchoscopy. Nevertheless, NIV provided superior oxygenation adequacy and stability in patients with a baseline PaO_2_ < 60 mmHg on room air, with more marked stability immediately and 30 min post-procedure. Vital parameters, including heart rate and respiratory rate, were also more stable with NIV than with HFT [18].

Regarding therapeutic procedures in which the FiO_2_ needs to be reduced to minimise the risk of fire during therapeutic flexible bronchoscopy under 40%, we identified only a single article addressing argon plasma and electrocautery performed via flexible bronchoscopy. This study reports a case series of 14 patients with relatively uncomplicated central airway lesions, considered to carry a low predictable risk of bleeding. Greater procedural safety was observed when these interventions were performed using non-invasive ventilation in CPAP mode with an FiO_2_ of 30%, compared with COT. No significant differences were noted in SpO_2_ levels or success rates, but improved haemodynamic control was achieved in terms of blood pressure and heart rate [44].

Therefore, NIV appears to provide superior oxygenation adequacy and cardiopulmonary parameter stability compared to HFT and COT in patients with moderate to severe hypoxaemia and a history of cardiac disease, provided PaO_2_/FiO_2_ > 150 mmHg.

## 4. Discussion

The respiratory support strategy should be individualised according to the patient’s type, risk factors, and the procedure to be performed. Some procedures (BAL, transbronchial biopsy, bronchial brushing, among others) can be carried out with extensive topical anaesthesia and superficial sedation. Others (EBUS, laser, etc.) may require deeper sedation, employing different pharmacological strategies including sedatives and analgesics. These drugs may alter the closing pressure of the upper airways, induce collapse, and affect both the respiratory pattern and ventilatory drive. Therefore, in cases of deep sedation, NIV may be necessary and preferable to other oxygenation strategies to ensure adequate ventilation and gas exchange.

Risk factors for respiratory failure following FB are not yet fully established. However, large retrospective analyses have identified several factors associated with adverse events, including extensive pulmonary infiltrates, PaO_2_ < 75 mmHg, SpO_2_ < 90%, and FEV_1_ < 1 L. In a series of 108 ventilated patients, Trouillet et al. [45] reported that the presence of acute respiratory distress syndrome (ARDS) and frequent procedural interruptions during FB were linked to hypoxaemic episodes. Similarly, Verra et al. observed that pre-procedural pulmonary function test results and radiographic findings were correlated with the lowest oxygen saturation recorded during bronchoscopy.

The European Society of Anaesthesiology (ESA) and the European Society of Intensive Care Medicine (ESICM) have issued guidelines regarding the use of non-invasive respiratory support in hypoxaemic patients during the perioperative and periprocedural periods. For hypoxaemic patients undergoing bronchoscopy, they recommend employing non-invasive respiratory support rather than COT, with a level of evidence of 2B. However, the guidelines do not provide specific recommendations on the choice between HFT, NIV, or CPAP.

HFT has been shown to be safe in most patients with mild to moderate ARF undergoing FB for diagnostic or therapeutic indications, including lung transplant recipients. In contrast, NIV provides more stable oxygenation during longer procedures or in patients with more severe ARF. Overall, HFT effectively enhances oxygenation in patients with lower oxygen demands, whereas NIV appears superior for those with severe ARF when compared with HFT.

A recent multicentre study including 142 patients at high risk of desaturation—such as those with morbid obesity, a narrow trachea, or baseline hypoxaemia/hypercapnia—compared HFT with COT and found that HFT significantly reduced the incidence of desaturation, procedural interruptions, and the need for escalated interventions during bronchoscopy [46].

Although the positive expiratory pressure generated by HFT diminishes substantially during oral breathing, potentially reducing its effects on alveolar distension and prevention of derecruitment, evidence indicates that HFT preserves oxygenation during BAL in FB by maintaining end-expiratory lung volume, preserving tidal volume, and reducing diaphragmatic effort compared with COT.

Bronchoscopy can induce cardiovascular alterations that may trigger adverse events in patients with underlying cardiac vulnerability. In such cases, both CPAP and NIV are recommended over HFT or COT as the preferred respiratory support strategies for patients with coexisting cardiac disease and/or heart failure. CPAP may be favoured over NIV in certain situations because it delivers a continuous flow that is simpler to implement; whereas both techniques maintain positive airway pressure throughout the respiratory cycle, NIV requires careful adjustment of ventilator settings to optimise patient–ventilator synchrony during inspiration.

Some interfaces for CPAP or NIV, including helmets and masks, have been specifically developed for use during FB, permitting bronchoscope insertion via the oral or nasal route. Nevertheless, air leaks around the interface or through the bronchoscope port may induce patient–ventilator asynchrony, which can be challenging to manage. The potential for microorganism dispersion should also be considered: when masks are well-fitted with minimal leaks, exhaled air disperses similarly to HFT, with reported distances of 172–332 mm. With helmet NIV, exhaled air escapes through the neck–helmet interface at 150–230 mm. By contrast, masks with intentional leaks via the exhalation port in single-limb circuits can produce exhaled air jets extending up to 916 mm [47].

NIV has emerged as a safe and effective modality during FB in patients with chronic respiratory conditions, particularly those with COPD or baseline hypercapnia. Unlike COT, which may deliver an inadequate FiO_2_ when the patient’s peak inspiratory flow surpasses the oxygen flow provided, NIV delivers positive airway pressure, thereby enhancing gas exchange, reducing the work of breathing, and preventing alveolar collapse during bronchoscopic procedures [48].

NIV not only ensures more effective maintenance of oxygenation during bronchoscopy but also mitigates CO_2_ retention, a clinically significant concern in hypoxaemic patients undergoing the procedure.

Randomised trials involving patients with acute exacerbations of COPD have shown that NIV is associated with lower rates of initial support failure and a reduced need for intubation compared with HFT (initial failure: 14% vs. 25%). Furthermore, a recent meta-analysis in hypercapnic COPD patients indicated that, while HFT and NIV demonstrated similar outcomes regarding mortality and intubation rates, the risk of treatment failure was significantly greater with HFT (RR 1.64; 95% CI: 1.04–2.60) [19].

The application of NIV during bronchoscopy appears to be the optimal strategy in patients with severe hypoxaemia or hypercapnia, such as those with advanced COPD. In summary, current evidence indicates that NIV in patients with chronic respiratory disease provides superior protection against both hypoxaemia and hypercapnia, lowers the risk of post-procedural ventilatory failure, and improves procedural safety compared with HFT or COT.

Currently, a multicentre, randomised, controlled clinical trial is in progress, enrolling 300 patients stratified according to the Horowitz index, to compare COT versus HFT, HFT versus NIV, and NIV versus invasive mechanical ventilation (IMV) [49].

**Figure 2:**
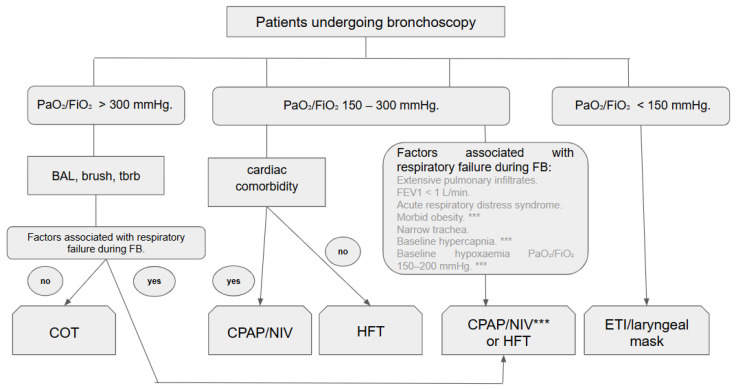
The algorithm for selection of respiratory support during flexible bronchoscopy. Abbreviations: PaO2/FIO2 (ratio of arterial oxygen pressure to inspired oxygen fraction), BAL (bronchoalveolar lavage), COT (conventional oxygen therapy), HFT (high-flow therapy), NIV (non-invasive ventilation), Tbrb (transbronchial biopsy), CPAP (continuous positive airway pressure), FEV1 (Forced Expiratory Volume in 1 s), ETI (endotracheal intubation), *** (in cases of morbid obesity, baseline hypercapnia, baseline hipoxaemia it would be highly recommended CPAP or NIV).

Based on the current literature, a possible approach is outlined in Figure 2.

Most studies have focused on the impact of oxygenation strategies on physiological parameters, with comparatively fewer addressing meaningful clinical outcomes. The quality of the reviewed literature is limited by small sample sizes and considerable heterogeneity among patient populations, precluding formal meta-analysis. Furthermore, most studies were conducted at single centres.

Moreover, there is considerable variability in the titration and settings of HFT, NIV, and CPAP, alongside marked differences in sedation protocols.

This review presents several limitations that warrant consideration. The literature search was confined to the PubMed and Cochrane databases, potentially omitting relevant studies indexed elsewhere. Given the limited number of rigorous trials directly comparing respiratory support strategies during flexible bronchoscopy, both randomised and high-quality observational studies were included. While this approach permitted a more comprehensive assessment of the available evidence, it may introduce a risk of bias. Furthermore, there remains a need for additional well-designed randomised controlled trials to facilitate direct comparisons among the various respiratory support modalities.

A key strength of this review lies in its current and comprehensive appraisal of the available evidence regarding diverse respiratory support strategies during bronchoscopy, rather than limiting the focus to comparisons between high-flow therapy and conventional oxygen therapy, which dominate existing literature. We conducted a comparative analysis of the three principal oxygen delivery modalities employed during bronchoscopy, examining their characteristics and potential advantages. This direct comparison offers a practical framework for clinicians in selecting the most appropriate oxygenation strategy for diagnostic bronchoscopy, as summarised in the algorithm presented in Figure 2, providing a rapid decision-making tool that is currently absent from the literature.

## 5. Conclusions

In conclusion, the selection of a respiratory support during flexible bronchoscopy should not be based on conventional oxygen therapy by default but must be guided by the type of procedure, the patient’s baseline status, and the anticipated risk of respiratory compromise. Consequently, the likelihood of respiratory deterioration during FB should be thoroughly assessed prior to the procedure [19]. Current evidence supports a structured, stepwise approach, as outlined in Figure 2.

For patients with mild to moderate oxygen requirements and no significant haemodynamic compromise, HFT is recommended over COT as it provides more stable oxygenation, reduces the incidence of desaturation, and minimises procedural interruptions.

In contrast, when bronchoscopy is undertaken in patients with more severe hypoxaemia, relevant cardiac comorbidities, or when deeper sedation is required, CPAP or NIV should be prioritised. Both modalities maintain positive airway pressure throughout the procedure, ensure adequate gas exchange, and contribute to greater cardiopulmonary stability.

Among patients with chronic respiratory disease, particularly COPD or hypercapnia, NIV offers further advantages over HFT and COT. These include superior protection against carbon dioxide retention, a lower risk of post-procedural ventilatory failure, and a reduced need for endotracheal intubation while maintaining more stable cardiopulmonary parameters. NIV unloads the respiratory muscles and counteracts dynamic hyperinflation, thereby preventing intubation and reducing in-hospital mortality in this group of patients [50,51].

Accordingly, the selection of respiratory support must be justified on clinical grounds: HFT is most appropriate for cases with mild to moderate oxygen demands, whereas CPAP or NIV should be reserved for patients with more severe respiratory or cardiac impairment or higher procedural risk. This stepwise framework, represented in Figure 2 and now detailed in the text, provides a pragmatic guide for clinicians to enhance safety and optimise outcomes during flexible bronchoscopy.

## Figures and Tables

**Table 1:** Main articles revised for this article: Abbreviations: ND: not defined. SpO_2_: oxygen saturation; PaO_2_: arterial oxygen pressure; PaCO_2_: arterial carbon dioxide pressure; HR: heart rate; BP: blood pressure; RR: respiratory rate; pH: measure of acidity or alkalinity in blood; ETI: endotracheal intubation; COT: conventional oxygen therapy; HFT: high-flow therapy; NIV: non-invasive ventilation; NIPPV: non-invasive positive pressure ventilation; CPAP: continuous positive airway pressure; PaO_2_/FiO_2_: ratio of arterial oxygen partial pressure (PaO2) to the fraction of inspired oxygen (Fi02), used as an index of oxygenation. LOS: Length of stay at hospital and intensive care unit.

Reference	No. of Patients	Study Type	Clinical Status	Support During FB	Main Objectives
Golpe, et al. [8]	44	Observational	ND	COT	SpO_2_
Longhini, et al. [9]	36	Randomised prospective	Chronic	COT vs. HFT	SpO_2_, PaO_2_
Zhang, et al. [10]	176	Randomised prospective	Acute	COT vs. HFT	SpO_2_, post-procedure respiratory support
Arias-Sánchez, et al. [11]	40	Observational	Acute	COT vs. HFT	SpO_2_
Quin H et al. [12]	142	Multicentre randomised prospective	Chronic	COT vs. HFT	SpO_2_, transcutaneous CO_2_, complications
Ben-Menachem, et al. [13]	76	Randomised prospective	Chronic	COT vs. HFT	SpO_2_
Maitre, et al. [14]	30	Randomised prospective	Acute	COT vs. CPAP	SpO_2_, PaO_2_, post-ventilatory support
Antonelli, et al. [15]	26	Randomised prospective	Acute	COT vs. NIPPV	PaO_2_/FiO_2_, HR, BP, ETI
Da Conceição et al. [16]	10	Prospective	Acute	NIPPV	SpO_2_, PaO_2_, PaCO_2_, ETI, mortality
Simon, et al. [17]	40	Randomised prospective	Acute	NIV vs. HFT	SpO_2_, PaO_2_/FiO_2_, PaCO_2_, HR, BP, procedure time, ETI, mortality
Saksitthichok, et al. [18]	51	Randomised prospective	Acute	NIV vs. HFT	SpO_2_, PaO_2_, PaCO_2_, pH, RR, HR, BP, dyspnoea
Tan, et al. [19]	225	Randomised, open-label, non-inferiority trial	Acute hypercapnia	HFT vs. NIV	ETI, blood gases, ICU/Hospital LOS, 28-day mortality

## Data Availability

No new data were created or analyzed in this study. Data sharing is not applicable to this article.

## Abbreviations

ARDS	Acute respiratory distress syndrome
ARF	Acute respiratory failure
BAL	Bronchoalveolar lavage
BP	Blood pressure
COPD	Chronic obstructive pulmonary disease
COT	Conventional oxygen therapy
CPAP	Continuous positive airway pressure
EBUS	Endobronchial ultrasound
ETI	Endotracheal intubation
FB	Flexible bronchoscopy
FEV_1_	Forced expiratory volume in one second
FiO_2_	Fraction of inspired oxygen
GOLD	Global Initiative for Chronic Obstructive Lung Disease
HFT	High-flow therapy
HR	Heart rate
ICU	Intensive care unit
IMV	Invasive mechanical ventilation
IPAP	Inspiratory positive airway pressure
LOS	Length of stay (hospital/ICU)
ND	Not defined
NIV	Non-invasive ventilation
NIPPV	Non-invasive positive pressure ventilation
OSA	Obstructive sleep apnoea
PaCO_2_	Partial pressure of carbon dioxide in arterial blood
PaO_2_	Partial pressure of oxygen in arterial blood
PaO_2_/FiO_2_	Ratio of arterial oxygen partial pressure to inspired oxygen fraction
PEEP	Positive end-expiratory pressure
RR	Respiratory rate
SpO_2_	Peripheral capillary oxygen saturation
